# Matrix attachment region combinations increase transgene expression in transfected Chinese hamster ovary cells

**DOI:** 10.1038/srep42805

**Published:** 2017-02-20

**Authors:** Chun-Peng Zhao, Xiao Guo, Si-Jia Chen, Chang-Zheng Li, Yun Yang, Jun-He Zhang, Shao-Nan Chen, Yan-Long Jia, Tian-Yun Wang

**Affiliations:** 1 Department of Biochemistry and Molecular Biology, Xinxiang Medical University, Henan 453003, China; 2 Pharmacy College, Xinxiang Medical University, Xinxiang 453003, Henan, China

## Abstract

Matrix attachment regions (MARs) are cis-acting DNA elements that can increase transgene expression levels in a CHO cell expression system. To investigate the effects of MAR combinations on transgene expression and the underlying regulatory mechanisms, we generated constructs in which the enhanced green fluorescent protein (eGFP) gene flanked by different combinations of human β-interferon and β-globin MAR (iMAR and gMAR, respectively), which was driven by the cytomegalovirus (CMV) or simian virus (SV) 40 promoter. These were transfected into CHO-K1 cells, which were screened with geneticin; eGFP expression was detected by flow cytometry. The presence of MAR elements increased transfection efficiency and transient and stably expression of eGFP expression under both promoters; the level was higher when the two MARs differed (i.e., iMAR and gMAR) under the CMV but not the SV40 promoter. For the latter, two gMARs showed the highest activity. We also found that MARs increased the ratio of stably transfected positive colonies. These results indicate that combining the CMV promoter with two different MAR elements or the SV40 promoter with two gMARs is effective for inducing high expression level and stability of transgenes.

Mammalian cell expression systems are widely used to express recombinant proteins for clinical applications owing to their capacity for post-translational modification and assembly of human protein-like molecular structures. Since their isolation in 1957^1^, Chinese hamster ovary (CHO) cells have been the preferred mammalian cell line for production of recombinant proteins for therapeutic applications^2^,^3^,^4^. The first clinically approved recombinant protein generated in CHO cells was tissue plasminogen activator^5^; since then, it is estimated that CHO cells have been used to produce >70% of therapeutic proteins in a global market valued at US $30 billion in annual sales^6^,^7^. Several lines were obtained from the initial clone of CHO cells; of these, the CНO-K1 line became the most commonly used^8^, and several sub-cell lines generated using specific expression technology have become industry standards, including CHO DHFR (dihydrofolate reductase system)^9^, CHO GS (glutamine synthetase)^10^, and CНO-DG44^11^.

The major problem regarding recombinant protein production in cultured cells is the extremely low transgene expression level. There are constant efforts to increase the yield of the target protein product by increasing cell culture density, and minimizing cell death and optimization of expression vector.

Transgene silencing and low expression levels are common problems in recombinant protein technology that result from positional effects related to neighboring chromatin^12^,^13^,^14^. Various regulatory elements have been used in order to enhance recombinant protein expression and stability. For example, matrix attachment regions (MARs) are genomic DNA sequences that serve as attachment points within the DNA to anchor chromatin to the nuclear matrix during interphase^15^. MARs have been shown to increase transgene expression levels as well as the proportion of positive colonies in CHO cell expression systems^16^,^17^,^18^,^19^,^20^,^21^.

We previously demonstrated that human β-interferon and β-globin MARs (iMAR and gMAR, respectively) used in combination more potently enhanced transgene expression as compared to two identical MARs^22^. However, the mechanism underlying this phenomenon is unclear. Other regulatory elements in the vector containing MARs can influence transgene expression. For instance, Ho *et al*. showed that combination of the SV40 promoter and MAR elements achieved both high expression level and stability^23^. In the present study, we investigated the effect of various combinations of iMAR and gMAR and simian virus (SV) 40 and cytomegalovirus (CMV) promoters on transgene expression in stably transfected CHO cells. Our findings provide a basis for the design of vectors for generating cell lines that have high and stable transgene expression.

## Results

### Transfection efficiency and transient expression of recombinant protein

We first evaluated effects of gMAR, iMAR, and their combination under the control of two different promoters on transgene expression in transfected CHO cells. CHO-K1 cells were transfected with the constructs and the transfection efficiency and transient eGFP expression were analyzed with an epifluorescence microscope 48 h later.

**Figure 1:**
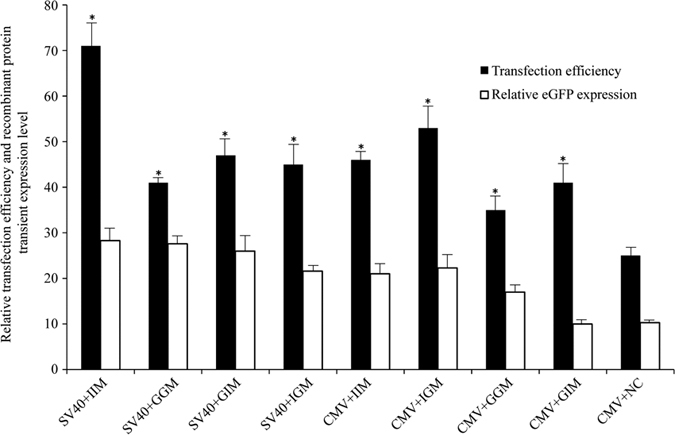
Transfection efficiency and recombinant protein transient expression of different MARs combined with different promoters in stably transfected pools. The eight constructed plasmids were transfected into CHO-K1 cells using Lipofectamine^®^ 2000 Transfection Reagent (Thermo Fisher Scientific), and the number of cell expressing the eGFP gene and MFI was determined after 48 h transfection; Three stably transfected pools were generated for each vector. Cells were collected and measured for the eGFP MFI with the FACS Calibur. The eGFP expression level value was normalized to the one obtained with the human CMV + NC, whose value was set to 10. These results are the mean values obtained for 3 independent experiments; Standard Error of Mean (SEM) is indicated (Student’s t test, *P < 0.05). SV40, simian virus 40 promoter; CMV, human cytomegalovirus IE gene promoter; IIM: β-interferon MAR + β-interferon MAR; GGM: β-globin MAR + β-globin MAR; GIM: β-globin MAR + β-interferon MAR; IGM: β-interferon MAR + β-globin MAR.

Transfection efficiency in CHO-K1 cells was higher for MAR-containing constructs as compared to the control vector (i.e., without MARs). Since all vectors used in this study were of approximately the same size, these results suggest that the presence of MARs improved transfection efficiency. Of the eight vectors, the transfection efficiency was the highest for iMAR + iMAR under SV40 promoter (71%). There were no differences in transfection efficiency between MAR vectors containing the SV40 vs. the CMV promoter (Fig. 1). We also observed that eGFP expression was higher in vectors with MARs, compared to those without them, with the exception of gMAR + iMAR under the CMV promoter. We set the lowest expression level as 10 (CMV+ NC under CMV promoter). The highest levels were observed for plasmids containing the SV40 promoter combined with iMAR, followed by those containing the SV40 promoter with gMAR + gMAR, gMAR + iMAR, and iMAR + gMAR. For vectors containing the CMV promoter and MARs, eGFP expression was lower than that for the same MARs under control of the SV40 promoter. The lowest expression level (i.e., less than that of the control vector) was associated with the construct harboring the CMV promoter along with gMAR + iMAR.

### Recombinant protein expression in stably transfected cell

**Figure 2:**
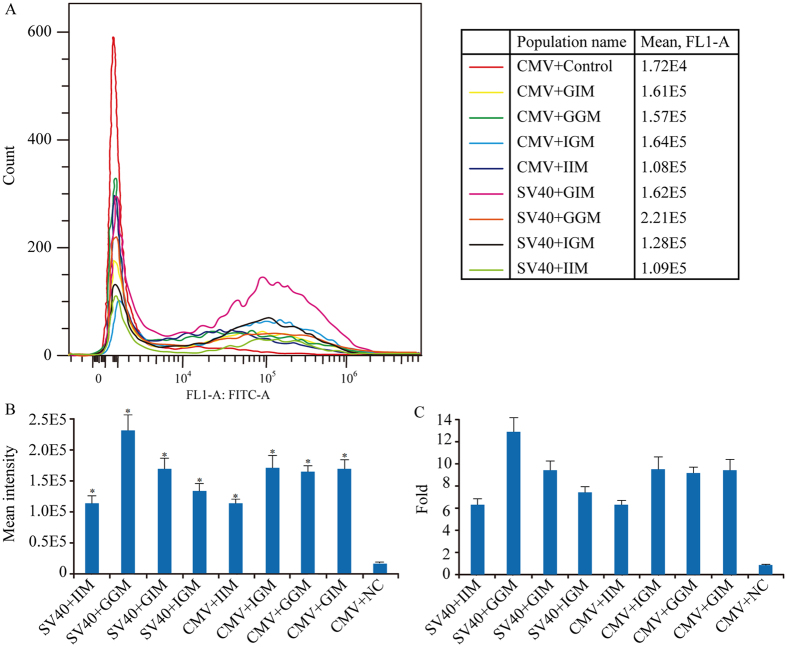
Effect of different MARs combination with different promoters on gene expression level in stably transfected pools. eGFP fluorescence profile was determined by cytometry for 100,000 polyclonal cells stably transfected with the reporter plasmid lacking a MAR element (no MAR), or containing the indicated MAR element. Cells were collected at day 15 and measured for the eGFP MFI with the FACS Calibur (**A**). Results were presented as the eGFP MFI normalized to those from the CMV promoter lacking MAR. Each value represents the average and standard deviation of three independent stably transfected pools (**B**) Fold statistical analysis results of expression level, and the eGFP MFI was normalized to CMV promoter lacking MARs (**C**). These results are the mean values obtained for 3 independent experiments, SEM is indicated (Student’s t test, *P < 0.05).

CHO-K1 cells were transfected with the vectors followed by drug selection to establish stable transfectants. The median fluorescence intensity (MFI) of eGFP was measured by flow cytometry. MARs increased eGFP expression in stably transfected cell lines with either the CMV or SV40 promoter relative to that in the control (Fig. 2). Among constructs containing the SV40 promoter, gMAR + gMAR induced the highest eGFP expression, followed by gMAR + iMAR, iMAR + gMAR, and iMAR + iMAR. SV40 with iMAR + iMAR induced higher transient but lower stable expression. For constructs containing the CMV promoter, iMAR + gMAR induced the highest eGFP expression, followed by gMAR + iMAR, gMAR + gMAR, and iMAR + iMAR (*P* < 0.05). Under both promoters, gMAR + gMAR, gMAR + iMAR, and iMAR + gMAR were associated with higher expression levels than iMAR + iMAR, indicating that these MAR combinations were more effective. SV40 with gMAR + gMAR induced the highest expression, which was 12.85-fold higher than that associated with the control vector (Fig. 2C).

### MARs increase the ratio of positive colonies

**Figure 3:**
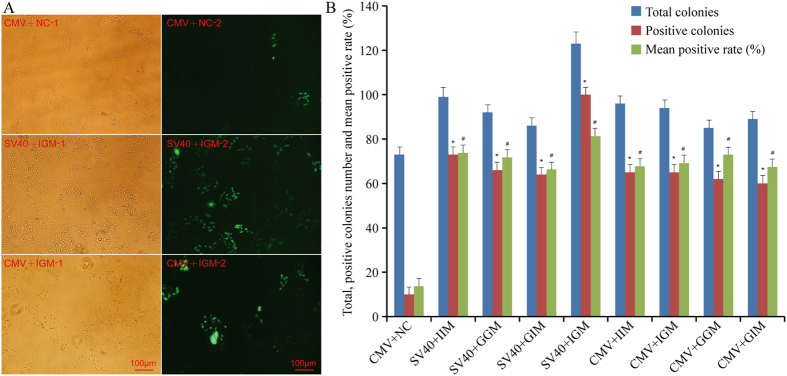
MAR increases the ratio of positive colonies and growth speed. CHO-K1 cells were added G418 (800 μg/ml) at 48 h after transfection cells were incubated for 14 days until single colonies appeared. The cell colonies number and size was observed and calculated based on either white light (left lane) and fluorescence (right lane) (**A**). Scale bars: 100 μm; (**B**) Statistical analysis result of the total, positive colonies number and mean positive ratio (%). These results are the mean values obtained for 3 independent experiments, SEM is indicated (Student’s t test, *,^#^*P* < 0.05).

Colonies of stably transfected CHO cells appeared after 2 weeks of drug selection. The number and growth characteristics of cell colonies were visualized by fluorescence microscopy (Fig. 3A). In cells transfected with vectors harboring MARs, the highest ratio of positive colonies (eGFP expression) was 81.30% (SV40 with iMAR + gMAR) as compared to 13.7% for cells transfected with the control vector (Fig. 3B). There was no significant difference in positive ratio between cells transfected with various MAR combinations, suggesting that MARs in general increase the efficiency of stable transfection. In addition, cells transfected with MARs grew more rapidly than those transfected with the control vector (Fig. 3B).

### Analysis of long-term recombinant protein expression stability

**Figure 4:**
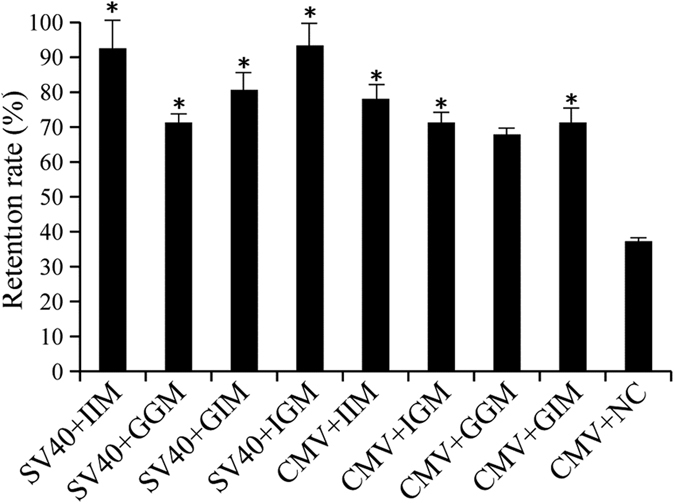
Retention of EGFP expression level of CHO cells transfected with different vector containing different MAR combination. The relative changes in eGFP expression was calculated as the ratio of eGFP MFI a clone measured at 30 generation of culture to the starting level for the same clone measured. These results are the mean values obtained for 3 independent experiments, SEM is indicated (Student’s t test, *P < 0.05).

We compared the stability of transgene expression of different MAR elements by evaluating the persistence of eGFP expression in CHO cells after 30 generation of culture. In all stably transfected cells, eGFP levels decreased gradually over time. The proportion of cells retaining eGFP expression was higher upon transfection with MAR-containing constructs than with the control vector. Colonies that maintain >70% of expression of the transfected transgene are considered to be stable^23^,^24^,^25^. According to this cutoff, seven of the eight cell lines transfected with MAR-containing vectors were stable after 30 generation of passaging. However, the retention rate of eGFP under the CMV and gMAR + gMAR combination was 67.82%. In contrast, the rate of retention of eGFP expressed from the control vector was only 36.97%. The results showed that the constructs with MARs combined with the SV40 promoter were more stable than those harboring the CMV promoter (Fig. 4).

## Discussion

The low and variable transgene expression in CHO cells is a major limitation to their use for production of recombinant proteins. MARs such as human gMAR and iMAR have been shown to be highly effective for enhancing the expression of transgenes—particularly those encoding proteins of therapeutic value—in stably transfected cells^16^,^17^,^20^.

CMV and SV40 are widely used promoters for recombinant protein expression in mammalian cells^26^. Both have high activity; however, the CMV promoter has more CG dinucleotides (31 as compared to six in the SV40 promoter^23^) and is thus susceptible to silencing by DNA methylation, which results in lowered productivity in long-term cultures^27^,^28^,^29^.

The present study investigated the ideal combination of CMV or SV40 promoter with gMAR and iMAR for maximal and stable transgene expression in CHO cells. We found that MARs increased the transfection efficiency and eGFP expression relative to those in the control vector, with SV40 promoter being superior to the CMV promoter. MARs have been shown to increase transgene expression in the CHO cell expression system; however, there are few reports on their effects on transfection efficiency and transient gene expression^18^,^30^. A previous study showed that one MAR from the mouse genome increased transient GFP and/or immunoglobulin expression from some but not all expression vectors, which was attributed to elements of the backbone vector^31^. We used the pEGFP-C1 backbone in the present study, which may have influenced the effect of the MARs on transient eGFP expression. Interestingly, we observed this effect with both CMV and SV40 promoters. Cells transfected with the construct harboring the SV40 promoter with gMAR + gMAR had higher levels of eGFP than those transfected with CMV promoter constructs, which is consistent with previously reported results^23^. The SV40 promoter with iMAR + gMAR did not show increased eGFP expression relative to other MAR combinations, which was in disagreement with another study^22^. This may be attributable to the fact that these investigators used a different backbone and only partial MAR sequences. In the previous studies the 1346~2074 position of iMAR and 904~1673 position of gMAR were used^22^,^32^, whereas we used full-length of iMAR + gMAR, Wang *et al*. investigated the effect of six β-globin MAR sub-fragments of gMAR on transgene expression in stably transfected CHO cells and found that various effect of these fragments with different transcription factor binding sites. Previous studies have shown that the SV40 promoter used in conjunction with MARs enhanced stable transgene expression^14^,^17^,^18^,^19^,^21^,^31^. In the present study, we found that iMAR + gMAR induced the highest eGFP expression under the control of the CMV promoter. Under both promoters, the combinations of gMAR + gMAR, gMAR + iMAR, and iMAR + gMAR showed a higher expression level than iMAR + iMAR. There have been studies on the effectiveness of CMV promoter with MARs, but these have reported conflicting findings^33^,^34^.

The stability analysis revealed that MARs increased the transgene retention rate as compared to cells transfected with the control vector without MARs; seven of eight stable cell lines transfected with MAR constructs retained >70% eGFP expression. In addition, we found that MARs increased the number of positive colonies, which also grew more rapidly under selection pressure than control-transfected cells.

In conclusion, our results show that MARs can increase transgene expression level, stability, and the rate of positive colonies. The ideal combination for generating CHO cell lines that have stable and high recombinant protein expression is the SV40 promoter combined with gMAR + gMAR, and SV40 promoter combined with iMAR + gMAR showed long-term recombinant protein expression stability and higher positive ratio of cell colonies. These findings can help to overcome the low and variable expression that presently limits the use of CHO cells.

## Materials and Methods

### Plasmids and constructs

**Figure 5:**
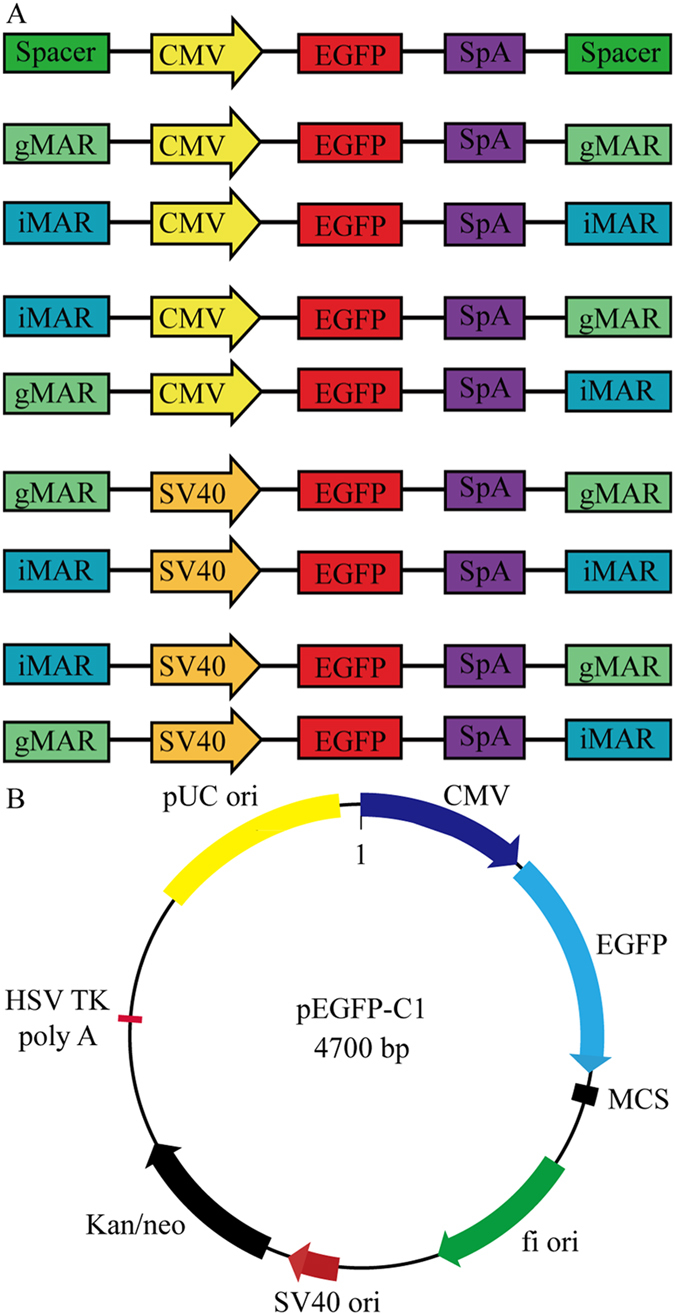
Schematic representation of vectors for evaluating the impact of different MARs combination on recombinant protein expression level and stability in CHO cells (**A**). Map of pEGFP-C1 used in this study (**B**). SpA, simian virus 40 early polyadenylation signal; eGFP, enhanced green fluorescence protein.

pEGFP-C1 containing the CMV promoter was used as the backbone vector in this study (Clontech, Mountain View, CA, USA). Vectors with or without MARs were constructed by replacing the CMV with the SV40 promoter and inserting iMAR or gMAR immediately upstream of the promoter and downstream of the SpA. The SV40 promoter was cloned from the pCAT3-control vector (Promega, Madison, WI, USA), and iMAR (GenBank access. no. M83137.1) and gMAR (GenBank access. no. L22754) were obtained by PCR amplification using the following primers: iMAR, 5′-ATCGGTACCAAGCTTCTGACAAATTATTCTTCCT-3′ and 5′-ATCGGTACCCAAAGGAGAAAAGTTTGTTGGCCTC-3′; and gMAR, 5′-ATCGGTACCGAATTCAGCAAGGTCGCCAC-3′ and 5′-TGAGGATCC CTATCAAGATATTTAAAGAAA-3′. The *utrophin* gene (GenBank access. no. NM_011682.4) was used as spacer DNA fragment according to a previous study^35^; a 2073-bp fragment (position 94–2166) was synthesized by General Biosystems (Chuzhou, China). In the control construct, MAR fragments were replaced with DNA sequences encoding a portion of the *utrophin* gene^24^. The PCR reaction was carried out using a kit (Takara Bio, Dalian, China) according to the manufacturer’s instructions. Restriction enzymes used for vector construction were purchased from New England Biolabs (Ipswich, MA, USA). Competent DH5α *Escherichia coli* cells used for cloning were purchased from Life Technologies (Carlsbad, CA, USA). Using pEGFP-C1 as the backbone, gMAR and/or iMAR was cloned into the 5′ and 3′ flanking regions of the expression cassette in each vector under the control of CMV or SV 40 promoter to construct nine vectors (Fig. 5).

### CHO cell culture

CHO-K1 cells (ATCC CCL-61) were cultured in Dulbecco’s modified Eagle’s medium (Gibco, Grand Island, NY, USA) supplemented with 10% heat-inactivated fetal bovine serum (Gibco) at 37 °C in a humidified incubator with 5% CO_2_.

### Generation of stably transfected cell lines

Cells were seeded into 6-well plates at approximately 3 × 10^6^ cells/well. Prior to transfection, plasmid DNA was linearized by digestion with *Apa*L restriction enzyme, which cleaves a unique site within the pUC ori of the plasmid. Cells were transfected with the above nine vectors using Lipofectamine 2000 reagent (Invitrogen, Carlsbad, CA, USA) according to the manufacturer’s instructions. After 48 h, 800 μg/ml geneticin (G418; Calbiochem, San Diego, CA, USA) was added to the culture medium and cells were incubated for 14 days until single colonies appeared. Those exhibiting stable transgene integration were cultured in the presence of 500 μg/ml G418 for 10–14 days, and then collected for further analysis.

### Flow cytometry

Enhanced green fluorescent protein (eGFP) expression was analyzed by flow cytometry using 1 × 10^4^–1 × 10^6^ CHO cells, with non-transfected cells used as the negative control. The results were analyzed with FlowJo software (Tree Star, Ashland, OR, USA). Three stably transfected pools were generated for each vector. To characterize each pool, 2 ml of culture at a density of 2 × 10^5^ cells/ml were seeded into each well of a 6-well plate after week 2 of G418 screening, and eGFP expression in the cells was analyzed with a FACS Calibur instrument (Becton Dickinson, Franklin Lakes, NJ, USA). A total of 100,000 fluorescent events were acquired using a 530/15 bandpass filter for the eGFP signal, which was obtained with fluorescence emission centered at 530 nm. The median fluorescence intensity (MFI) of each vector was also measured.

### Analysis of long-term recombinant protein expression stability

CHO cells stably transfected with the vectors were maintained as cultures. The MFI for each vector type was measured by flow cytometry and the stability of eGFP expression from each vector was calculated as the ratio of MFI at the end of 30 generation of culture to that at the start of testing.

### Statistical analysis

Data ware analyzed using SPSS v.18.0 software (SPSS Inc., Chicago, IL, USA), and are reported as mean ± standard deviation. Differences between groups were analyzed by single-factor analysis of variance, and the t test was used for pairwise comparisons. Differences with P values < 0.05 were considered statistically significant.

## Additional Information

**How to cite this article**: Zhao, C.-P. *et al*. Matrix attachment region combinations increase transgene expression in transfected Chinese hamster ovary cells. *Sci. Rep.* **7**, 42805; doi: 10.1038/srep42805 (2017).

**Publisher's note:** Springer Nature remains neutral with regard to jurisdictional claims in published maps and institutional affiliations.
